# Software Application Profile: *dynamicLM*—a tool for performing dynamic risk prediction using a landmark supermodel for survival data under competing risks

**DOI:** 10.1093/ije/dyad122

**Published:** 2023-09-06

**Authors:** Anya H Fries, Eunji Choi, Julie T Wu, Justin H Lee, Victoria Y Ding, Robert J Huang, Su-Ying Liang, Heather A Wakelee, Lynne R Wilkens, Iona Cheng, Summer S Han

**Affiliations:** Quantitative Sciences Unit, Department of Medicine, Stanford University School of Medicine, Stanford, CA, USA; Quantitative Sciences Unit, Department of Medicine, Stanford University School of Medicine, Stanford, CA, USA; Division of Oncology, Department of Medicine, Stanford University School of Medicine, Stanford, CA, USA; Quantitative Sciences Unit, Department of Medicine, Stanford University School of Medicine, Stanford, CA, USA; Quantitative Sciences Unit, Department of Medicine, Stanford University School of Medicine, Stanford, CA, USA; Division of Gastroenterology and Hepatology, Department of Medicine, Stanford University School of Medicine, Stanford, CA, USA; Palo Alto Medical Foundation Research Institute, Palo Alto Medical Foundation, Palo Alto, CA, USA; Division of Oncology, Department of Medicine, Stanford University School of Medicine, Stanford, CA, USA; Stanford Cancer Institute, Stanford, CA, USA; Cancer Epidemiology Program, University of Hawaii Cancer Center, Honolulu, HI, USA; Department of Epidemiology and Biostatistics, University of California, San Francisco, CA, USA; Quantitative Sciences Unit, Department of Medicine, Stanford University School of Medicine, Stanford, CA, USA; Stanford Cancer Institute, Stanford, CA, USA; Department of Epidemiology and Population Health, Stanford University School of Medicine, Stanford, CA, USA; Department of Neurosurgery, Stanford University School of Medicine, Stanford, CA, USA

**Keywords:** Landmark, competing risks, dynamic prediction, time-dependent variables, R

## Abstract

**Motivation:**

Providing a dynamic assessment of prognosis is essential for improved personalized medicine. The landmark model for survival data provides a potentially powerful solution to the dynamic prediction of disease progression. However, a general framework and a flexible implementation of the model that incorporates various outcomes, such as competing events, have been lacking. We present an R package, *dynamicLM*, a user-friendly tool for the landmark model for the dynamic prediction of survival data under competing risks, which includes various functions for data preparation, model development, prediction and evaluation of predictive performance.

**Implementation:**

*dynamicLM* as an R package.

**General features:**

The package includes options for incorporating time-varying covariates, capturing time-dependent effects of predictors and fitting a cause-specific landmark model for time-to-event data with or without competing risks. Tools for evaluating the prediction performance include time-dependent area under the ROC curve, Brier Score and calibration.

**Availability:**

Available on GitHub [https://github.com/thehanlab/dynamicLM].

## Introduction

Accurate prediction of disease prognosis is essential for effective clinical decision-making.^1^ Traditional prediction models are commonly used to estimate the risk of the event of interest (e.g. mortality) at a fixed time point—such as at the time of diagnosis^2^^,^^3^ or the end of curative treatment^4^^,^^5^—and thus may fail to provide updated risk estimates that can change over time.^6^^,^^7^ Temporal changes in patient data can affect the subsequent risk, and incorporating these changes can help update risk estimations for optimal patient management.^6^^,^^8^

The landmark model^6^^,^^8–10^ and joint modeling^11^^,^^12^ are two approaches used for dynamic prediction for survival data. Joint modeling can provide accurate estimates by simultaneously modeling the longitudinal markers and time-to-event outcome, but it is computationally intensive and requires more modelling assumptions. Alternatively the landmark model, built based on the concept of ongoing risk assessment times (i.e. landmarks) following the baseline, reduces the computational burden to provide updated risk estimates for large-scale data. Specifically, the dataset is transformed into multiple censored datasets based on a prediction window of interest and predefined landmarks. A model is then fitted on this stacked dataset (i.e. supermodel) that incorporates the dynamic trajectories of the patients, which can be used to provide the most up-to-date risk predictions.

Most landmark models for dynamic prediction have been applied in the context of standard survival outcomes using the Cox model.^6^^,^^9^^,^^10^ However, it is often observed that other causes of failure (i.e. competing events) may preclude the occurrence of the event of interest. Ignoring competing risks from prediction can lead to an overestimation of the risk.^13–15^ The cause-specific Cox (CSC) model^16^ is one solution that combines several fitted Cox models to avoid the overestimation of the predicted risk.^15^ There have been efforts to incorporate competing risks into dynamic prediction,^10^^,^^14^^,^^17^^,^^18^ but they lack a user-friendly implementation. Furthermore, practical evaluations (e.g. computational feasibility) of the dynamic landmark model for competing-risk data have been lacking.

We introduce an R package, *dynamicLM*, a tool to implement the landmark model for dynamic predictions using survival data incorporating competing risks. The package provides a framework including data preparation, model fitting, prediction, evaluation and visualization. We illustrate this method by applying it to predict second primary lung cancer risk from the Multiethnic Cohort Study^19^ with a set of simulations.

## Implementation

The *dynamicLM* tool provides a simple framework to perform dynamic w-year risk predictions, i.e. predicting the risk of developing the event of interest within w years from each risk assessment. Risk prediction for the next w years is made at baseline (e.g. diagnosis) and at a later set of risk assessment times (‘landmark’ times).

Input data can take various forms; covariates can be static (e.g. race), time-varying (e.g. biomarker changes) or a mix. Time-varying covariates can be in long or wide formats. Outcomes can be time-to-event for one cause (standard survival data) or a specific event in the presence of competing event(s). *dynamicLM* includes implementations of the Cox landmark model for standard survival data and the CSC landmark model for competing risk data.

The hazard of each cause-specific landmark supermodel for cause j (j=1, …, C) from a landmark time $s\in \left[s_{0},s_{L}\right]$ for time *t* (s≤t≤s+w) is:


$$h_{j}\left(t|Z\left(s\right),s\right)=h_{j0}\left(t\right)\exp\left(\theta_{j}\left(s\right)+\beta_{j}\left(s\right)Z(s)\right)$$

where $\theta \left(s\right)$ models the main effects of the landmark time s and Z(s) denotes the most recent covariates of individual observed until s. In particular, the baseline hazard at time t, $h_{j0}\left(t\right)\exp\left(\theta_{j}\left(s\right)\right)$, is the probability that a person with all zero covariates will experience the event in the next instant if that person survives to t from s. The interaction of s with the covariates, modelled by $\beta_{j}\left(s\right)$, captures the time-dependent effects of covariates. The w-year survival and (cause-specific) cumulative incidence for cause j can be predicted at any point s in the window $\left[s_{0},s_{L}\right]$:


$$S\left(s+w|Z\left(s\right),s\right)=\exp\left(-\int_{s}^{s+w}\sum_{j=1}^{C}h_{j}\left(t|Z\left(s\right),s\right)dt\right)$$

and


$$F_{j}\left(s+w|Z\left(s\right),s\right)=\int_{s}^{s+w}h_{j}\left(t|Z\left(s\right),s\right)S\left(t|Z\left(s\right),s\right)dt$$

respectively.

Estimating cumulative incidence in *dynamicLM* involves an iteration over the causes and landmarks in the super dataset. It also uses *prodlim,*^20^*riskRegression,*^21^*survival*^22^ and *dynpred*^23^ R packages. The *dynamicLM* package has a PDF manual that is downloadable from GitHub [https://github.com/thehanlab/dynamicLM] and accessible in R program (See Supplementary Method 1, available as Supplementary data at *IJE* online).

## Use

This section presents the application of *dynamicLM* to develop a risk-prediction model for second primary lung cancer (SPLC) among lung cancer patients using the CSC landmark supermodel. The data contain 3844 ever-smoking patients diagnosed with initial primary lung cancer (IPLC) between 1993 and 2007, followed through 2017, in the Multiethnic Cohort Study (MEC) (Supplementary Method 2, available as Supplementary data at *IJE* online).

The event of interest is the time from IPLC to SPLC diagnosis (Cause 1) with competing events of lung cancer death (Cause 2) and other-cause death (Cause 3). First, we load the package and data into our R session:

> devtools::install_github("thehanlab/dynamicLM")

> library(dynamicLM)

> data(splc)

*The example dataset in this R package is synthetic because the original MEC data are only available under a data use agreement. Readers can apply the code but cannot replicate the same estimates.

The super dataset setup requires: (ii) the outcome columns; and (ii) variable types (fixed vs time-varying). Fixed variables (e.g. sex) do not change over time, whereas time-varying covariates (e.g. smoking status at baseline and 10-year follow-up surveys) do. In this example, the fixed covariates include age at IPLC diagnosis (‘age.ix’), family history of lung cancer (‘fh’), prior history of cancer (‘ph’), IPLC stage (‘stage.ix’), IPLC histology and IPLC treatment. The time-varying covariates include smoking-related variables. The following code sets this up:

> outcome <- list(time = "Time", status = "event")

> fix_covs <- c("age.ix", "male", "fh", "ph",          "bmi", "stage.ix",

         "hist_AD", "hist_LC",          "hist_NSCLC_NOS", "hist_SC",

         "hist_OTH", "surgery.ix",          "radiation.ix",

         "chemo.ix", "quityears")

> vary_covs <- c("smkstatus", "cigday",           "packyears")

> covs <- list(fixed = fix_covs,          varying = vary_covs)

To predict the 5-year SPLC risk during the first 3 years of IPLC diagnosis, we first specified a prediction window and established a set of landmarks (i.e. risk assessment time points at 0, 1, 2 and 3 years from IPLC diagnosis), producing one stacked dataset (‘lmdata’) of four landmark datasets (Supplementary Figure S1 and Supplementary Table S1, available as Supplementary data at *IJE* online). The original dataset (splc) is in long-form format that may include multiple observations per patient if the patient has 10-year follow-up survey data (Supplementary Method 2, available as Supplementary data at *IJE* online). The column ‘T.fup’ represents the duration between baseline and follow-up measurements of time-varying covariates (‘smkstatus’, ‘cigday’ and ‘packyears’). This follow-up time is specified in the ‘rtime’ argument:

> w <- 5           #5-year prediction                 window

> lms <- seq(0, 3, by = 1) #landmarks

> lmdata <- stack_data(data = splc,              outcome = outcome,              lms = lms,

                 w = w, covs = covs,                id = "ID",

               format = "long",                rtime = "T.fup")

> table(lmdata$data$LM) #Number of patients per landmark

Certain time-varying covariates can be manually updated as time passes. In our data, smoking quit years (‘quityears’) increase linearly for former smokers:

> former_smokers <- lmdata$data$smkstatus == 2

> lmdata$data[former_smokers, "quityears"] <-

    lmdata$data[former_smokers,     "quityears"] +

    lmdata$data[former_smokers, "LM"]

The next step involves creating; (i) interaction terms between chosen covariates (‘lm_covs’) and landmark times (linear, quadratic, or other forms using ‘func_covars’) to examine time-dependent effects of the covariate and check the proportional hazard assumption; and (ii) transformations of the landmark time variables (‘func_lms’).

Unlike time-varying covariates, time-dependent effects occur when the hazard of a covariate changes over time. For example, the effect of radiotherapy given to treat IPLC on SPLC risk has been reported to increase over time.^24^ We selected a priori a list of variables and associated transformations (linear, quadratic) to be checked for the time-dependent effects based on domain knowledge of how variable effects change over time. Certain other transformations (e.g. logarithmic) can be added if necessary (Supplementary Method 2, available as Supplementary data at *IJE* online). The newly created interaction terms are identified by each variable name followed by an underscore and number (e.g. stage.ix_1 = stage.ix*LM, and stage.ix_2 = stage.ix*LM^2^).

We add linear and quadratic landmark time transformations using ‘func_lms’ to incorporate the smoothing across landmarks into modelling:

> lm_covs <- c("packyears", "radiation.ix",            "ph", "stage.ix",

         "hist_AD", "hist_LC",          "hist_NSCLC_NOS", "hist_SC",

         "hist_OTH")

> lmdata <- add_interactions            (lmdata = lmdata,             lm_covs = lm_covs,

            func_covars = c("linear",             "quadratic"),

            func_lms = c("linear",             "quadratic"))

> colnames(lmdata$data) #columns of added terms

A model is then fit on the stacked dataset. Our model selection procedure is in Supplementary Method 2, available as Supplementary data at *IJE* online:

formula <- "Hist(Time, event, LM) ∼      male + ph + ph_1 +     radiation.ix + radiation.ix_1 +      packyears + quityears +      hist_AD + hist_LC +      hist_NSCLC_NOS +      hist_OTH + hist_SC +      hist_AD_1 + hist_LC_1 +      hist_NSCLC_NOS_1 +      hist_OTH_1 + hist_SC_1 +      stage.ix + stage.ix_1 + stage.ix_2 +      LM_1 + LM_2 +      cluster(ID)"

> supermodel <- dynamic_lm(lmdata = lmdata,

        formula = as.formula(formula),

        type = "CSC")

> print(supermodel)

The dynamic cause-specific hazard ratios of the final model are depicted in Supplementary Table S2 (available as Supplementary data at *IJE* online). Covariates with time-dependent effects included IPLC radiotherapy (Supplementary Figure S2, available as Supplementary data at *IJE* online). To plot dynamic hazard ratios to capture the increased effect on SPLC risk:

> plot(supermodel, "radiation.ix", logHR = FALSE)

Individual 5-year predicted risk can be estimated:

> p <- predict(supermodel); summary(p$preds)

**Figure 1.**
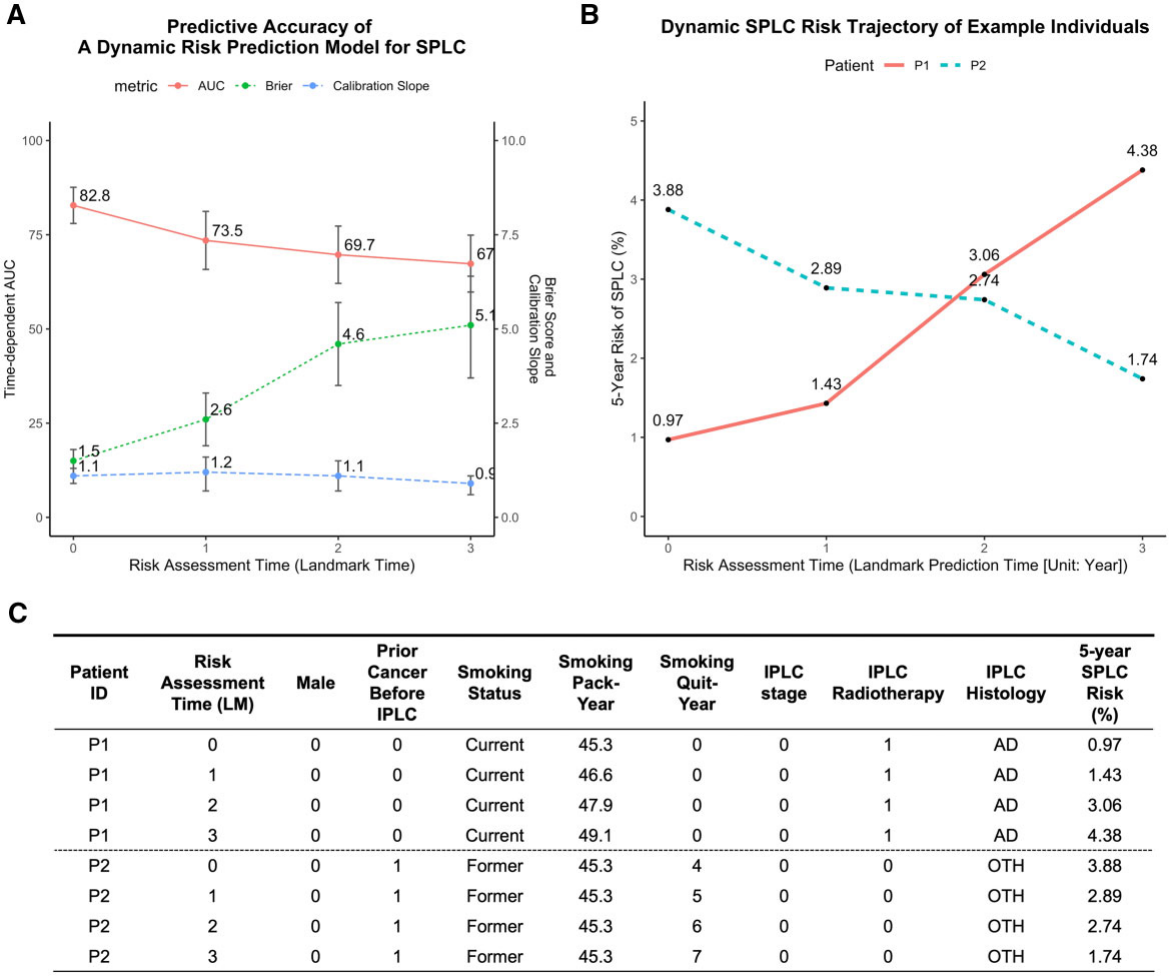
A dynamic prediction model for 5-year risk of SPLC among IPLC patients in the Multiethnic Cohort Study. (A) Predictive performance [discrimination (AUC), calibration (calibration slope) and predictive accuracy [Brier score]) of the proposed model across risk assessment times. (B) The dynamic trajectory of estimated 5-year SPLC risk across different risk assessment times (landmark times) among two example individuals. With a 5-year predefined prediction window (w = 5), the risk of developing SPLC in 5 years was estimated at each landmark time [i.e. risk assessment time or prediction time point (unit: years)] across the horizontal coordinates. In this example, the 5-year risk of developing SPLC in Patient P1 is 0.97% when predicted at IPLC diagnosis (Landmark = 0), which is updated to 1.43% [when predicted at 1 year after IPLC diagnosis (Landmark = 1)], 3.06% [when predicted at 2 years after IPLC diagnosis (Landmark = 2)] and 4.38% [when predicted at 3 years after IPLC diagnosis (Landmark = 3)] at successive landmarks based on the most recent patient information seen till each landmark. (C) In Panel (B), Patient P1’s 5-year SPLC risk increases over the landmark times due to accumulated smoking pack-years and IPLC radiotherapy which have time-dependent effects for SPLC risk; Patient P2’s 5-year SPLC risk decreases over time due to increasing smoking quit-years and ‘other’ IPLC histology which have time-dependent effects for SPLC risk (see Supplementary Table S2, available as Supplementary data at *IJE* online). AD, adenocarcinoma IPLC histology; AUC, srea under the receiver operating characteristic curve; ID, identifier; IPLC, initial primary lung cancer; LM, landmark time; OTH, Other (not belonging to non-small-cell lung cancer nor small-cell lung cancer) IPLC histology; SPLC, second primary lung cancer

To evaluate the model, we create calibration plots and compute area under the curve (AUC) and Brier score. Bootstrapped internal validation and external validation can be performed (Supplementary Method 2, available as Supplementary data at *IJE* online). Our model showed good calibration (calibration slope: 0.9–1.2 across landmarks) (Figure 1; Supplementary Figure S3, available as Supplementary data at *IJE* online) and high discrimination at baseline (AUC >80%). The AUC decreased over landmarks because most of the data in model fitting come from the baseline in MEC, which could be one limitation of this data. If patient features are updated over time, the model’s predictive accuracy could stay consistent:

> par(mfrow = c(2, 2))

> cal <- calplot(list("CSC" = p),

          xlim = c(0, 0.25),           ylim = c(0, 0.25),

          method= "quantile",           q = 10) #deciles

> score(list("CSC" = p)) #AUC and Brier

An example of two patients’ dynamic 5-year SPLC risk is shown in Figure 1B. The increasing SPLC risk in Patient P1 was driven by latent IPLC radiotherapy, which has an increasing effect on SPLC over time (Figure 1C):

> par(mfrow = c(1, 1))

> inds <- lmdata$data[lmdata$data$ID %in%             c("ID2", "ID7"), ]

> plotrisk(supermodel, inds, format = "long",        ylim = c(0, 0.2))

## Simulation

We conducted simulations to evaluate the computational feasibility of *dynamicLM* and to assess the impact of accounting for competing risks in the landmark supermodel when they are present. The simulation methods are included in Supplementary Method 3 (available as Supplementary data at *IJE* online).

The computational feasibility of *dynamicLM* compared with joint modeling (packages *JMbayes2*^12^ and *FastJM*^25^) across different scenarios is shown in Supplementary Table S3 (available as Supplementary data at *IJE* online). *dynamicLM* has the shortest mean time (<0.1 s) for fitting a CSC landmark model and calculating individual-level predictions (<41 s). *JMbayes2* takes considerably longer (>240 s for model fitting and >120 s for prediction using 3000 individuals). Although *FastJM* scales better than *JMbayes2* for model fitting, it cannot handle multiple time-varying covariates. Landmark modelling (*dynamicLM*) is scalable for high-dimensional data with multiple time-varying covariates.

**Figure 2.**
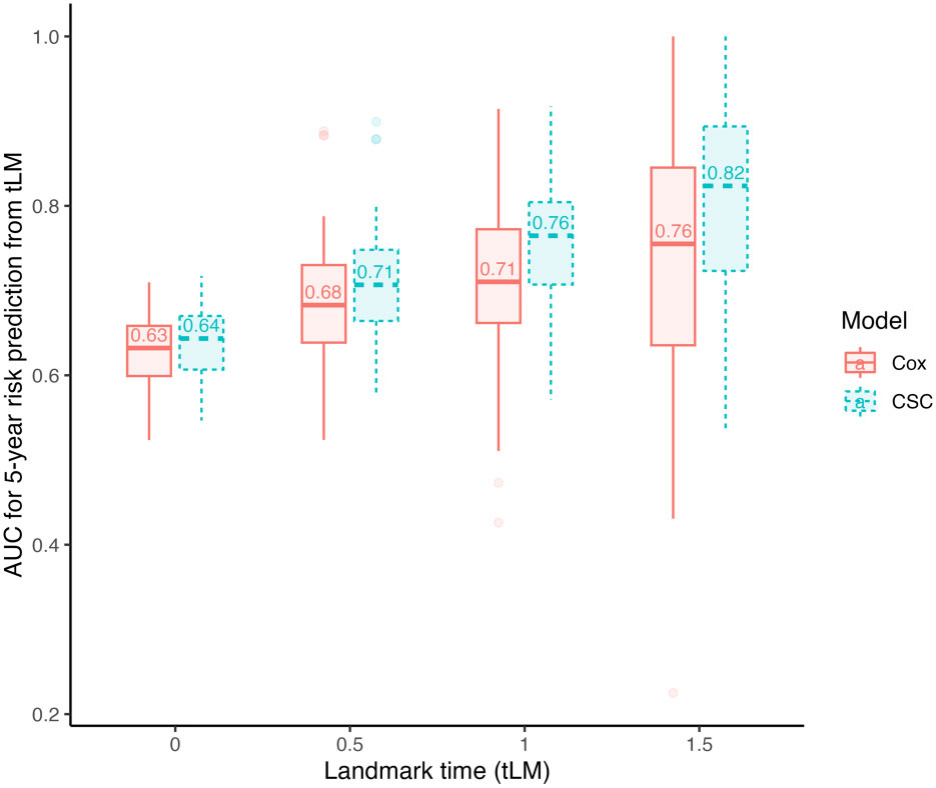
Simulation study to evaluate the impact of model misspecification for analysing landmark superdata under competing risks. Dynamic time-dependent AUCs are compared across four different landmark times of 0, 0.5, 1 and 1.5 years from baseline for predicting a 2-year risk using a standard Cox landmark model (that censors competing events) vs a cause-specific Cox (CSC) landmark model (correct model that accounts for competing risks). We simulated 100 datasets in the presence of competing risks, with each dataset including 1000 individuals and 10 predictors. The details of the simulation methods are shown in the Supplementary Methods (available as Supplementary data at *IJE* online). AUC, area under the receiver operating characteristic curve; CSC, cause-specific Cox model; tLM, landmark time

The simulation results for evaluating the impact of correctly handling competing events on predictive accuracy are shown in Figure 2. Using a standard Cox landmark model instead of a CSC landmark model leads to reduced predictive performance when competing risks exist. The difference in AUC between the two models becomes more pronounced over landmark times.

Additionally, we compared the two models in our application example of SPLC (vs simulation). The predicted risk score was overestimated (1.5–5.3 times higher) in a standard Cox landmark vs CSC landmark (Supplementary Figure S4, available as Supplementary data at *IJE* online), emphasizing the importance of correctly handling competing risks in dynamic prediction.

## Discussion

In this study, we presented the R package *dynamicLM* that implements a flexible framework for building a dynamic landmark supermodel for competing risk data, covering the entire pipeline for data preparation, model development, prediction and evaluation. By providing researchers with practical tools and instructions on how and when to use this approach, *dynamicLM* holds great promise to improve individual risk predictions by using updated patient data.

The proposed implementation can be applied in many clinical settings, such as predicting a cancer recurrence^6^ (or a therapy response) using time-varying biomarkers (i.e. circulating tumour DNA)^1^ in clinical trials or predicting second malignancies using updated patients’ treatment histories in electronic health records. Future research directions include incorporating regularization for feature selection in high-dimensional data and developing unified performance metrics summarized across landmarks to evaluate the predictive performance of the landmark model.

## Ethics approval

Study participants provided informed consent in accordance with the Declaration of Helsinki and the study was approved by the institutional review boards of the University of Hawaii and the University of Southern California.

## Supplementary Material

dyad122_Supplementary_Data

## Data Availability

The data underlying this analysis were provided by the Multiethnic Cohort Study (MEC) under data use agreement. Researchers interested in the MEC data may submit an inquiry online: [https://www.uhcancercenter.org/for-researchers/mec-data-sharing].

## References

[1] Kurtz DM , Esfahani MS, Scherer F et al Dynamic risk profiling using serial tumor biomarkers for personalized outcome prediction. Cell 2019;178:699–713.e619. 31280963 10.1016/j.cell.2019.06.011 PMC7380118

[2] Han SS , Rivera GA, Tammemägi MC et al Risk stratification for second primary lung cancer. J Clin Oncol 2017;35:2893–99. 28644772 10.1200/JCO.2017.72.4203 PMC5946715

[3] Barcenas CH , Song J, Murthy RK et al Prognostic model for De Novo and recurrent metastatic breast cancer. JCO Clin Cancer Inform 2021;5:789–804. 34351787 10.1200/CCI.21.00020 PMC8807018

[4] D'Journo XB , Boulate D, Fourdrain A et al; International Esodata Study Group. Risk prediction model of 90-day mortality after esophagectomy for cancer. JAMA Surg 2021;156:836–45. 34160587 10.1001/jamasurg.2021.2376 PMC8223144

[5] Jones GD , Brandt WS, Shen R et al A genomic-pathologic annotated risk model to predict recurrence in early-stage lung adenocarcinoma. JAMA Surg 2021;156:e205601. 33355651 10.1001/jamasurg.2020.5601 PMC7758824

[6] Fontein DBY , Klinten Grand M, Nortier JWR et al Dynamic prediction in breast cancer: proving feasibility in clinical practice using the TEAM trial. Ann Oncol 2015;26:1254–62. 25862439 10.1093/annonc/mdv146

[7] Luo SJ , Choi E, Aredo JV et al Smoking cessation after lung cancer diagnosis and the risk of second primary lung cancer: the multiethnic cohort study. JNCI Cancer Spectr 2021;5:pkab076. 10.1093/jncics/pkab076 PMC8487318 34611582

[8] van Houwelingen H , Putter H. Dynamic Prediction in Clinical Survival Analysis. Boca Laton, FL: CRC Press, 2011.

[9] Paige E , Barrett J, Stevens D et al Landmark models for optimizing the use of repeated measurements of risk factors in electronic health records to predict future disease risk. Am J Epidemiol 2018;187:1530–38. 29584812 10.1093/aje/kwy018 PMC6030927

[10] Yang Z , Hou Y, Lyu J, Liu D, Chen Z. Dynamic prediction and prognostic analysis of patients with cervical cancer: a landmarking analysis approach. Ann Epidemiol 2020;44:45–51. 32220511 10.1016/j.annepidem.2020.01.009

[11] Asar Ö , Ritchie J, Kalra PA, Diggle PJ. Joint modelling of repeated measurement and time-to-event data: an introductory tutorial. Int J Epidemiol 2015;44:334–44. 25604450 10.1093/ije/dyu262

[12] Rizopoulos D , Papageorgiou G, Miranda Afonso P. *JMbayes2: Extended Joint Models for Longitudinal and Time-To-Event Data*. R package version 0.2–4. 2022. https://cran.r-project.org/package=JMbayes2 (30 September 2022, date last accessed).

[13] Leroy T , Monnet E, Guerzider S et al Let us not underestimate the long-term risk of SPLC after surgical resection of NSCLC. Lung Cancer 2019;137:23–30. 31521979 10.1016/j.lungcan.2019.09.001

[14] Nicolaie MA , van Houwelingen JC, de Witte TM, Putter H. Dynamic prediction by landmarking in competing risks. Stat Med 2013;32:2031–47. 23086627 10.1002/sim.5665

[15] Varadhan R , Weiss CO, Segal JB et al Evaluating health outcomes in the presence of competing risks: a review of statistical methods and clinical applications. Med Care 2010;48:S96–105. 20473207 10.1097/MLR.0b013e3181d99107

[16] Benichou J , Gail MH. Estimates of absolute cause-specific risk in cohort studies. Biometrics 1990;46:813–26. 2242416

[17] He P , Eriksson F, Scheike TH, Zhang MJ. A proportional hazards regression model for the sub-distribution with covariates adjusted censoring weight for competing risks data. Scand Stat Theory Appl 2016;43:103–22. 27034534 10.1111/sjos.12167 PMC4809648

[18] Cortese G , Gerds TA, Andersen PK. Comparing predictions among competing risks models with time-dependent covariates. Stat Med 2013;32:3089–101. 23494745 10.1002/sim.5773 PMC3702649

[19] Choi E , Sanyal N, Ding VY et al Development and validation of a risk prediction model for second primary lung cancer. J Natl Cancer Inst 2022;114:87–96. 34255071 10.1093/jnci/djab138 PMC8755509

[20] Gerds TA. *prodlim: Product-Limit Estimation for Censored Event History Analysis*. R package version 2019.11.13. 2019. https://CRAN.R-project.org/package=prodlim (30 September 2022, date last accessed).

[21] Gerds TA , Kattan MW. Medical Risk Prediction Models: With Ties to Machine Learning. Boca Laton, FL: CRC Press, 2021.

[22] Therneau T. *A Package for Survival Analysis in R*. R package version 3.2–13. 2021. https://CRAN.R-project.org/package=survival (30 September 2022, date last accessed).

[23] Putter H. *dynpred: Companion Package to “Dynamic Prediction in Clinical Survival Analysis”.* R package version 0.1.2. 2015. https://CRAN.R-project.org/package=dynpred (30 September 2022, date last accessed).

[24] Choi E , Lam VT, Aredo JA et al Abstract 3445: Long term effect of radiotherapy on risk of second primary lung cancer and overall mortality among lung cancer patients. Cancer Res 2022;82:3445.

[25] Li S , Li N, Wang H et al Efficient algorithms and implementation of a semiparametric joint model for longitudinal and competing risk data: with applications to massive biobank data. Comput Math Methods Med 2022;2022:1362913. 35178111 10.1155/2022/1362913 PMC8846996

