# Supplementary material for: Software Application Profile: dynamicLM—a tool for performing dynamic risk prediction using a landmark supermodel for survival data under competing risks
Source: Int J Epidemiol. 2023 Sep 6;52(6):1984–9. doi: 10.1093/ije/dyad122 (PMC10749764; doi:10.1093/ije/dyad122)
Supplement: dyad122_Supplementary_Data [file dyad122_supplementary_data.zip › dyad122_Supplementary_Data/ije-2022-10-1197-File004.docx]

# **Supplementary Materials**

CONTENTS

1. **Supplementary Tables**

**Supplementary Table S1**. An example of the stacked super dataset

**Supplementary Table S2**. The dynamic prediction model for 5-year risk of developing SPLC among ever-smoking IPLC patients with time-constant and time-varying covariates

**Supplementary Table S3**. Mean time (in seconds) for model fitting and prediction using a cause-specific cox landmark supermodel and joint model under varying sample sizes and numbers of fixed and time-varying predictors.

1. **Supplementary Figures**

**Supplementary Figure S1**. Number of patients at risk and number of patients with SPLC in the landmark datasets over time since the time at IPLC diagnosis in Multiethnic Cohort (MEC)

**Supplementary Figure S2**. Time-varying cause-specific hazard ratios (csHR) for SPLC over time since the time at IPLC diagnosis in Multiethnic Cohort (MEC)

**Supplementary Figure S3**. Calibration of a dynamic prediction model for a 5-year SPLC risk in the presence of competing risks of death in the landmark datasets over time since the time at IPLC diagnosis

**Supplementary Figure S4**. Estimated 5-year SPLC risk among IPLC patients using competing-risk cause-specific Cox landmark supermodel vs. regular Cox landmark supermodel across different landmark times (i.e., risk assessment times) since the time at IPLC diagnosis in Multiethnic Cohort (MEC)

1. **Supplementary Methods**
2. The *dynamicLM* package
3. Details in SPLC model development using a cause-specific Cox (CSC) landmark model
4. Simulation methods
5. Landmark model
   1. Overview
   2. Landmarking
   3. Sliding landmark model and extension to competing risks
   4. The landmark supermodel
   5. Prediction
   6. Model performance
6. **References**

# **1. Supplementary Tables**

**Supplementary Table S1.** An example of the stacked super dataset

| ID | Time | event^b^ | prior  _cancer | IPLC  _stage | IPLCtx  _rad | IPLCtx  _chemo | IPLCtx  _surg | age.at.  assessment | smoking  _status | cigs  /day | pack  _years | quit  _years | LM^c^ |
| --- | --- | --- | --- | --- | --- | --- | --- | --- | --- | --- | --- | --- | --- |
| 1 | 1.51 | 2 | 1 | 1 | 1 | 0 | 0 | 77 | Former | 15.5 | 31.80 | 0.5 | 0 |
| 1 | 1.51 | 2 | 1 | 1 | 1 | 0 | 0 | 78 | Former | 15.5 | 31.80 | 1.5 | 1 |
| 2 | 5.00^a^ | 0 | 1 | 0 | 0 | 1 | 1 | 80 | Former | 25.5 | 45.30 | 18 | 0 |
| 2 | 6.00^a^ | 0 | 1 | 0 | 0 | 1 | 1 | 81 | Former | 25.5 | 45.30 | 19 | 1 |
| 2 | 7.00^a^ | 0 | 1 | 0 | 0 | 1 | 1 | 82 | Former | 25.5 | 45.30 | 20 | 2 |
| 2 | 8.00^a^ | 0 | 1 | 0 | 0 | 1 | 1 | 83 | Former | 25.5 | 45.30 | 21 | 3 |
| 3 | 3.09 | 2 | 0 | 1 | 0 | 0 | 0 | 71 | Current | 25.5 | 32.50 | 0 | 0 |
| 3 | 3.09 | 2 | 0 | 1 | 0 | 0 | 0 | 72 | Current | 25.5 | 33.78 | 0 | 1 |
| 3 | 3.09 | 2 | 0 | 1 | 0 | 0 | 0 | 73 | Current | 25.5 | 35.05 | 0 | 2 |
| 3 | 3.09 | 2 | 0 | 1 | 0 | 0 | 0 | 74 | Current | 25.5 | 36.33 | 0 | 3 |
| 4 | 1.75 | 3 | 1 | 1 | 0 | 0 | 0 | 71 | Current | 15.5 | 27.50 | 0 | 0 |
| 4 | 1.75 | 3 | 1 | 1 | 0 | 0 | 0 | 72 | Current | 15.5 | 28.28 | 0 | 1 |

Abbreviation: IPLC: initial primary lung cancer, LM: landmark time.

^a^ Because prediction time window is 5-year from each risk assessment time (landmark prediction time), individuals who are free of event up to 5 years of follow-up from the assessment time (LM) are censored.

^b^ Event = 1 (SPLC); Event = 2 (lung cancer death); Event = 3 (other-cause death); Event = 0 (Censored)

^c^ Landmark time (LM) refers the risk assessment time.

**Supplementary Table S2.** The dynamic prediction model for 5-year risk of developing SPLC among ever-smoking IPLC patients with time-constant and time-varying covariates in Multiethnic Cohort (MEC)

|  | Adjusted Cause-Specific  Hazard Ratio | (95% CI) | P value |
| --- | --- | --- | --- |
| **Covariates with time-constant effects** | | | |
| Male | 2.17 | (1.25-3.76) | 0.005 |
| Smoking pack-year^a^ | 1.01 | (1.00-1.02) | 0.048 |
| Smoking quit-year^a^ | 0.97 | (0.94-1.01) | 0.118 |
|  |  |  |  |
| **Covariates with time-dependent effects**^b,c^ | | | |
| IPLC radiotherapy |  |  |  |
| Constant effect | 0.51 | (0.24-1.09) | 0.086 |
| Time-varying effect (LM)^b^ | 1.29 | (0.96-1.72) | 0.086 |
| Prior history of cancer |  |  |  |
| Constant effect | 2.59 | (1.51-4.41) | <.001 |
| Time-varying effect (LM)^b^ | 0.84 | (0.69-1.02) | 0.075 |
| Advanced stage of IPLC^d^ |  |  |  |
| Constant effect | 0.45 | (0.17-1.19) | 0.109 |
| Time-varying effect (LM)^b^ | 2.80 | (1.28-6.14) | 0.001 |
| Time-varying effect (LM^2^)^b^ | 0.67 | (0.49-0.91) | 0.012 |
| Histology of IPLC^e^ |  |  |  |
| Constant effect |  |  |  |
| Adenocarcinoma | 0.87 | (0.45-1.66) | 0.68 |
| Large cell | 4.67 | (1.97-11.06) | <.001 |
| NSCLC/NOS | 0.71 | (0.16-3.16) | 0.659 |
| Small cell | 0.22 | (0.03-1.38) | 0.108 |
| Other | 0.85 | (0.31-2.35) | 0.757 |
| Time-varying effect (LM)^b^ |  |  |  |
| Adenocarcinoma | 0.96 | (0.78-1.19) | 0.754 |
| Large cell | 0.74 | (0.55-1.01) | 0.058 |
| NSCLC/NOS | 0.81 | (0.45-1.45) | 0.491 |
| Small cell | 2.09 | (1.34-3.25) | 0.001 |
| Other | 0.61 | (0.40-0.91) | 0.017 |
|  |  |  |  |
| **Effects of landmark time (in years)** | | | |
| Linear effect (LM) | 1.08 | (0.90-1.30) | 0.367 |
| Quadratic effect (LM^2^) | 1.01 | (0.97-1.04) | 0.777 |

Abbreviation: IPLC: initial primary lung cancer, LM: landmark time, SPLC: second primary lung cancer

^a^ Time-varying covariates whose values changed during follow-up

^b^ To test for time-dependent effects of the features, interactions between each selected covariate and landmark time (LM) variable (both linear and quadratic) were included in the model. Interactions that nullify significance of the main covariate were removed; only significant linear and quadratic interactions were retained.

^c^ Specific hazard ratios (HR) for covariates having time-dependent effect are calculated by the following formula: HR = [constant effect x (Time-varying effect)^LM^].

^d^ Reference = Early stage of IPLC

^e^ Reference = Squamous cell carcinoma

**Supplementary Table S3.** Mean time (in seconds) for model fitting and prediction using a cause-specific cox landmark supermodel and joint model under varying sample sizes and numbers of fixed and time-varying predictors

| # covariates | | **Model fitting time (s)** | | | **Prediction time (s)** | | |
| --- | --- | --- | --- | --- | --- | --- | --- |
| Fixed | Varying | *dynamicLM* | *JMbayes2* | *FastJM* | *dynamicLM* | *JMbayes2* | *FastJM* |
| **n = 500** | | | | | | | |
| 1 | 1 | 0.0307 | 36.7 | 0.561 | 1.6 | 25.0 | 270.6 |
| 1 | 3 | 0.0241 | 84.8 | - | 1.4 | 35.7 | - |
| 1 | 5 | 0.0254 | 130.9 | - | 1.5 | 51.8 | - |
| 10 | 1 | 0.0293 | 38.3 | 0.801 | 1.6 | 23.9 | 261.8 |
| 10 | 3 | 0.0381 | 142.6 | - | 1.9 | 45.6 | - |
| 10 | 5 | 0.0375 | 194.9 | - | 1.9 | 63.8 | - |
| 20 | 1 | 0.0317 | 82.4 | 0.908 | 1.7 | 24.2 | 244.8 |
| 20 | 3 | 0.0325 | 173.0 | - | 1.6 | 36.4 | - |
| 20 | 5 | 0.0328 | 217.0 | - | 1.6 | 51.5 | - |
| **n = 1000** | | | | | | | |
| 1 | 1 | 0.0330 | 64.1 | 0.931 | 5.2 | 38.7 | 456.4 |
| 1 | 3 | 0.0344 | 172.7 | - | 5.6 | 63.7 | - |
| 1 | 5 | 0.0358 | 265.8 | - | 5.6 | 94.5 | - |
| 10 | 1 | 0.0383 | 93.2 | 1.360 | 5.6 | 40.5 | 477.8 |
| 10 | 3 | 0.0421 | 223.4 | - | 5.7 | 65.2 | - |
| 10 | 5 | 0.0417 | 315.6 | - | 5.7 | 96.4 | - |
| 20 | 1 | 0.0478 | 160.6 | 1.416 | 5.7 | 42.3 | 481.5 |
| 20 | 3 | 0.0485 | 351.3 | - | 5.9 | 67.4 | - |
| 20 | 5 | 0.0511 | 917.5 | - | 5.9 | 145.5 | - |
| **n = 3000** | | | | | | | |
| 1 | 1 | 0.0644 | 243.3 | * | 38.6 | 120.5 | * |
| 1 | 3 | 0.0743 | 607.0 | - | 40.9 | 209.1 | - |
| 1 | 5 | 0.0855 | 932.9 | - | 40.7 | 322.6 | - |
| 10 | 1 | 0.1138 | 339.0 | * | 41.2 | 126.6 | * |
| 10 | 3 | 0.1221 | 815.5 | - | 42.9 | 219.2 | - |
| 10 | 5 | 0.1117 | 1109.3 | - | 41.1 | 326.5 | - |
| 20 | 1 | 0.1256 | 548.3 | * | 41.4 | 131.7 | * |
| 20 | 3 | 0.1319 | 1170.9 | - | 42.1 | 221.1 | - |
| 20 | 5 | 0.1573 | 1518.4 | - | 41.7 | 337.7 | - |

Specifications: Simulations were run on a 2021 MacBook Pro in RStudio with R version 4.2.1

- Cannot compute: FastJM cannot handle multiple time-varying covariates

* Cannot compute due to error message: E step ran into issue for the Xth subject. Program stops.

Note: We generated 20 datasets for each sample size *n* and a varying number of fixed and time-varying terms in the model. For the landmark supermodel, model fitting was conducted across four landmark times (0, 0.5, 1, 1.5). Predictions were made for the primary cause of interest at baseline (landmark time 0) for all individuals. The details of the simulation methods are shown in **Supplementary Methods**.

# **2. Supplementary Figures**

**Supplementary Figure S1.** Number of patients at risk (A) and number of patients with SPLC (B) in the landmark datasets over time since the time at IPLC diagnosis in Multiethnic Cohort (MEC)

**A**

**B**


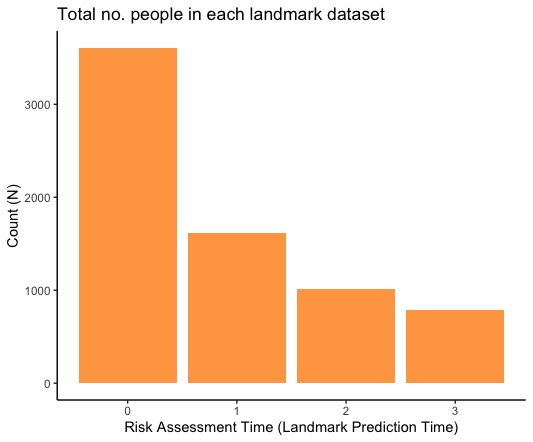

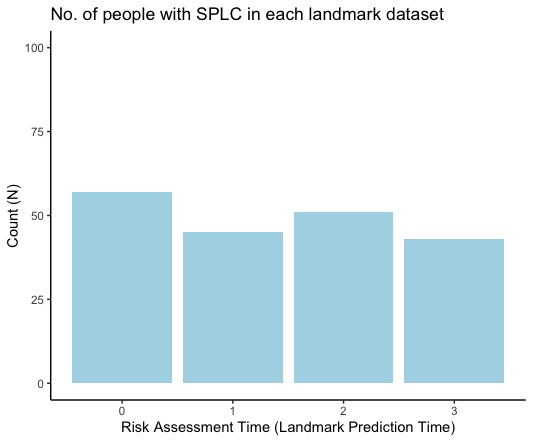


**Abbreviations:** LM: landmark time; SPLC: second primary lung cancer.

**Supplementary Figure S2.** Time-varying cause-specific hazard ratios (csHR) for SPLC over time since the time at IPLC diagnosis in Multiethnic Cohort (MEC)

Time-dependent effects of the selected model features were examined by adding the terms for interactions between each feature and landmark time variable (both linear and quadratic). The csHR of covariates with significant time-dependent effect (p-value <.1) was plotted (See **Supplementary Table S1**). In the first panel (“Prior history of cancer”), having a prior history of cancer was positively associated with the risk of developing SPLC at the time of IPLC diagnosis (i.e., study baseline [Landmark prediction time = 0]). However, the effect of having a prior history of cancer was diminished at successive landmarks (i.e., when assessed at 0.5 and 1 years after IPLC diagnosis), and became nonsignificant from landmark =1.5 (i.e., when assessed 1.5 years after IPLC diagnosis) and later. This time-dependent changes in the effect of the selected model features is critical for accurately providing updated prediction risk across different risk assessment time points after baseline.

**
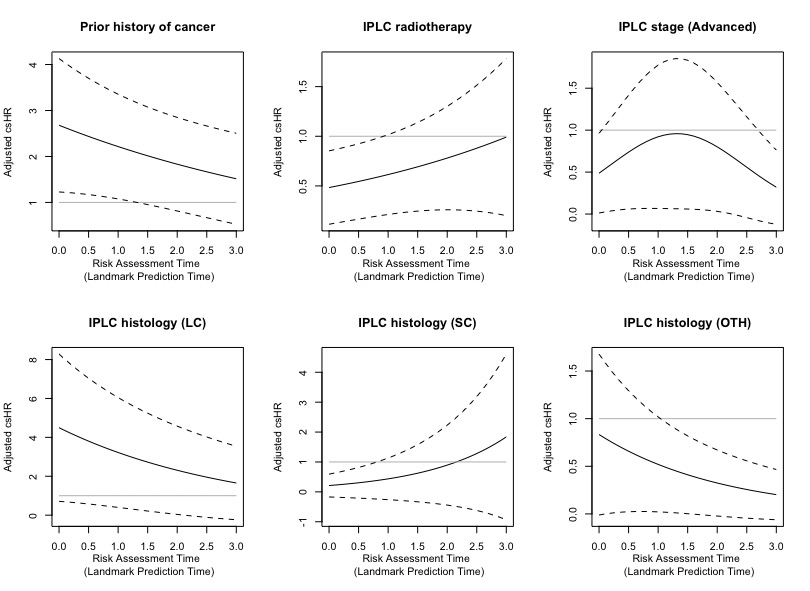
**

**Abbreviation:** csHR, cause-specific hazard ratio; IPLC, initial primary lung cancer; LC, large cell; LM, landmark; SC, small cell.

**Supplementary Figure S3.** Calibration of a dynamic prediction model for a 5-year SPLC risk in the presence of competing risks of death in the landmark datasets over time since the time at IPLC diagnosis in Multiethnic Cohort (MEC)


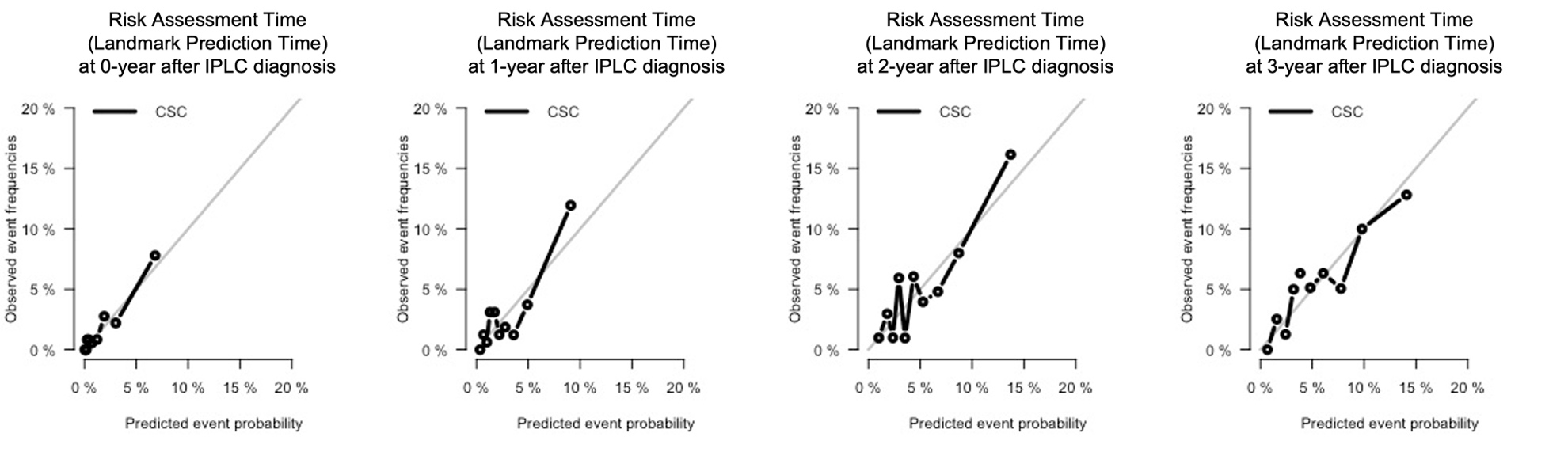


**Abbreviations:** IPLC: initial primary lung cancer; LM: landmark time (risk assessment time); SPLC: second primary lung cancer.

**Supplementary Figure S4.** Estimated 5-year SPLC risk among IPLC patients using competing-risk cause-specific Cox landmark supermodel vs. regular Cox landmark supermodel across different landmark times (i.e., risk assessment times) since the time at IPLC diagnosis in Multiethnic Cohort (MEC)*


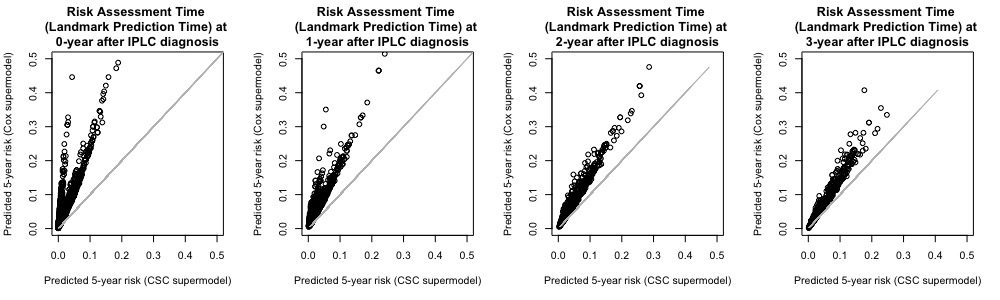


**Abbreviation:** CSC: cause-specific Cox model, IPLC: initial primary lung cancer, SPLC: second primary lung cancer.

***Note**: The MEC data in this example contains 3,332 ever-smoking patients who were diagnosed with IPLC between 1993 and 2007 and followed up for SPLC through 2017. The event of interest is the time from IPLC diagnosis to SPLC incidence (cause 1); two competing events that can occur before developing SPLC include lung cancer death (cause 2) and other-cause death (cause 3). In the regular Cox landmark supermodel, all competing events were considered as censored, while cause-specific Cox landmark supermodel fit several Cox landmark models for each outcome (outcome of interest and competing events) to estimate the unbiased predicted risk of the event.

# **3. Supplementary Methods**

**[1] The *dynamicLM* package**

A vignette help file for dynamicLM that includes comprehensive descriptions about the role of each function, parameters in each function, and interpretation of output values is (1) downloadable as a PDF manual from GitHub (<https://github.com/thehanlab/dynamicLM>), (2) accessible online (<https://github.com/thehanlab/dynamicLM/blob/8e80cddcd3e3c35bf130096c6b210ed28b866362/man/dynamicLM_0.3.0.pdf>), or (3) available by typing the following in R ?function_name. The following is a screen capture of the first page of the PDF manual.


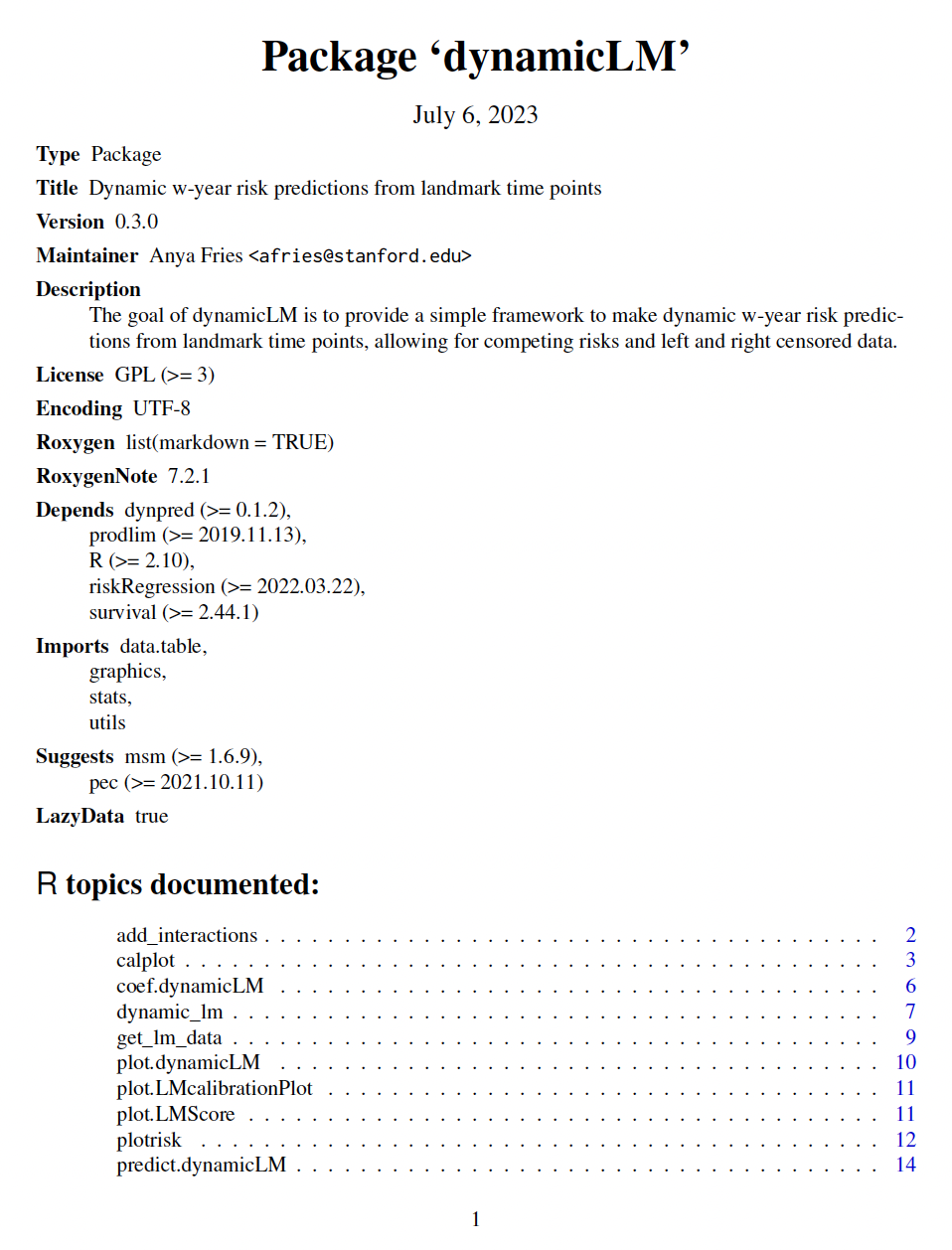


## **[2] Details in SPLC model development using a cause-specific Cox (CSC) landmark model**

- - - 1. **Study cohort**

This example dataset contains 3,332 ever-smoking patients who are part of the Multiethnic Cohort Study (MEC),^1,2^ who were diagnosed with initial primary lung cancer (IPLC) between 1993 and 2007 and followed up for SPLC through 2017. SPLC cases were identified via linkage to the SEER registries. Patients who did not incur any event at the last follow-up date (Dec/31/2017) were censored. SPLC was ascertained based on clinical criteria by Martini and Melamed^3^: if the subsequent tumor is diagnosed two years after IPLC diagnosis or if it has different histology. Comprehensive baseline information was collected at cohort enrollment (1993-1996) through a self-reported questionnaire; updated smoking-related variables were available through a 10-year follow-up survey (2003-2008) among 21.1% of the study cohort (n=704). If the 10-year follow-up information was obtained before or at the time of IPLC diagnosis, we utilized the updated data as baseline information; if the follow-up data was collected after IPLC diagnosis, we used both baseline and the follow-up values to capture the time-varying covariate changes after IPLC diagnosis. The event of interest is the time from IPLC diagnosis to SPLC incidence (cause 1); two competing events that can occur before developing SPLC include lung cancer death (cause 2) and other-cause death (cause 3). First, we load the package and data into our R session.

- devtools::install_github("thehanlab/dynamicLM")
- library(dynamicLM)
- data(splc)

(*Note: The dataset in this R package is synthetic because the MEC data is only available under a data use agreement. Readers can apply the code but cannot replicate the same estimates or draw clinical findings.)

- - - 1. **Two types of variables: fixed vs. time-varying covariates**

The super dataset setup first requires: (1) the outcome columns and (2) variable types (fixed vs. time-varying). Both fixed and time-varying variables were considered as candidate predictors for model development. Static (fixed) variables included: sex (“male”), prior history of cancer before IPLC diagnosis (“ph”), IPLC stage (“stage.ix”), IPLC histology (“hist_AD”, “hist_LC”,”hist_NSCLC_NOS”, “hist_SC”, “hist_OTH”), first course treatment (“surgery.ix”, “radiation.ix”, “chemo.ix”). Time-varying covariates—for which status or values can change over time after baseline (i.e., IPLC diagnosis)—included the following: smoking status (“smkstatus”), smoking pack-year (“packyears”), and cigs/day (“cigday”) collected at 10-year follow-up survey, and smoking quit-year with automatic update by year).

The categorical variables are coded as shown below:

“male”: 1 = male, 0 = female

“ph”: 1 = prior history of cancer, 0 = no prior history of cancer

“stage.ix”: 1 = advanced-stage (IV), 0 = early-stage (I-III)

“hist_AD”: 1 = adenocarcinoma IPLC, 0 = histology other than adenocarcinoma

“hist_LC”: 1 = large cell IPLC, 0 = histology other than large cell

“hist_NSCLC_NOS”: 1 = non-small cell lung cancer/not otherwise specified (NSCLC/NOS), 0 = 0 = histology other than NSCLC/NOS

“hist_SC”: 1 = small cell lung cancer (SCLC), 0 = histology other than small cell

“hist_OTH”: 1 = other (non NSCLC nor SCLC) histology, 0 = NSCLC or SCLC

“surgery.ix”: 1 = had surgery for IPLC treatment, 0 = had no surgery

“radiation.ix”: 1 = had radiotherapy for IPLC treatment, 0 = had no radiotherapy

“chemo.ix”: 1 = had chemotherapy for IPLC treatment, 0 = had no chemotherapy

“smkstatus”: 2 = former smokers, 3 = current smokers

- outcome <- list(time = "Time", status = "event")
- fix_covs <- c("age.ix", "male", "fh", "ph", "bmi", "stage.ix",

"hist_AD", "hist_LC", "hist_NSCLC_NOS", "hist_SC", "hist_OTH",

"surgery.ix", "radiation.ix", "chemo.ix", "quityears")

- vary_covs <- c("smkstatus", "cigday", "packyears")
- covs <- list(fixed = fix_covs, varying = vary_covs)
  - - 1. **Landmark setting to create a stacked dataset**

To predict 5-year risk of SPLC at any assessment time points between 0 and 3 years after IPLC diagnosis, we must set landmark timepoints, a prediction window, and a data format. The landmarks and prediction time window (“w”) can be determined based on data availability as well as the reasonable time window that the baseline exposure can impact the outcome of interest. We set landmarks (“lms”) every year between 0 and 3 years (0, 1, 2, and 3 years) from IPLC diagnosis.

The data format is specified in the “format” argument. The original dataset (splc) is in long-form format that may include multiple observations of a patient if the patient has 10-year follow-up survey data. The column “T.fup” represents the 10-year follow-up time, i.e., the duration between baseline and 10-year follow-up measurements of time-varying covariates ( “smkstatus”, “cigday”, and “packyears”). This 10-year follow-up time is specified in the “rtime” argument.

One stacked dataset (“lmdata”) is generated from landmark datasets created at the given landmarks (**Supplementary Figure S1**). The values of time-varying covariates are updated (if available) across landmark datasets. The stacked data column “LM” indicates each landmark. An example of the stacked dataset created for this analysis is shown in **Supplemental Table S1**.

- w <- 5 # 5-year prediction window
- lms <- seq(0, 3, by = 1)# landmarks
- lmdata <- stack_data(data = splc, outcome = outcome, lms = lms, w = w,

covs = covs, format = "long", rtime = "T.fup", id = "ID")

- table(lmdata$data$LM) # number of patients per landmark dataset (Corresponding to Supp. Fig 1A, but please keep in mind that the example dataset (splc) in this R package is synthetic because the MEC data is only available under a data use agreement. Readers can apply the code but cannot replicate the same estimates.)

Certain time-varying covariates can be automatically updated as time passes. In our data, quit years increases linearly for former smokers.

- former_smokers <- lmdata$data$smkstatus == 2
- lmdata$data[former_smokers, "quityears"] <-

lmdata$data[former_smokers, "quityears"] +

lmdata$data[former_smokers, "LM"]

- - - 1. **Creating interaction terms to capture time-dependent effects and to integrate the smoothing effect**

This stage involves creating 1) interaction terms between the selected covariates (“covars”) and landmark times (linear, quadratic, or other forms using func_covars) to examine time-dependent effects of covariates, and 2) the linear and quadratic landmark time variables (“func_LMs” to incorporate the smoothing effect over the stacked landmark datasets in model-fitting.

Unlike time-varying covariates, time-dependent effects occur when the hazard of a variable on the risk of SPLC changes over time. For example, the risk of radiotherapy given to treat IPLC has been reported to increase over time.^4^ This step is important in terms of capturing the true effect of covariates on the outcome as well as checking the proportional hazard assumption. If there is a significant interaction term between covariates and landmark times, then the proportional hazards assumption is violated (i.e., the hazard ratio varies over landmark times). It is thus necessary to include both the main and interaction terms. Please refer to section (e) for the method to identify time-dependent effects.

In the current SPLC model development, we selected a priori a list of variables (i.e., IPLC radiotherapy, IPLC stage, IPLC histology, prior history of cancer, and smoking pack-years) to be checked for the time-dependent effects based on literature (but readers may want to test all available covariates); the two forms of transformation (linear and quadratic) of landmark times were selected based on domain knowledge of how variable effects change over time. Commonly, the time-dependent effect of a variable can be captured in a linear or quadratic form, but certain other transformations (i.e., logarithmic) can be added if necessary.

The newly created interaction terms are identified by variable name followed by underbar and number (i.e., stage.ix_1 = stage.ix x LM, and stage.ix_2 = stage.ix x LM^2^).

- # A priori variables to be examined for time-dependent effect
- interaction_covs <- c("packyears", "radiation.ix", "ph", "stage.ix",

"hist_AD", "hist_LC", "hist_NSCLC_NOS", "hist_SC", "hist_OTH")

- lmdata <- add_interactions(lmdata = lmdata, lm_covs = lm_covs,

func_covars = c("linear", "quadratic"),

func_lms = c("linear", "quadratic"))

- colnames(lmdata$data) # Check the newly created variables
  - - 1. **Identifying variables having time-dependent effects on SPLC risk**

After creating the interaction terms, we identify the covariates with significant time-dependent effects. To examine the time-dependent effect of a certain variable (e.g., “packyears”), we fit a model with the main covariate (“packyears”) and interaction terms (“packyears_1”, “packyears _2” for LM and LM^2^), as shown in the example code below. If adding interactions nullifies the significance of the main covariate, we consider the covariate to have no time-dependent effects over landmark times, satisfying the proportional hazard assumption.

If the main covariate remains significant after adding interaction terms, we examined the significance of the interaction terms to retain only the significant interactions. If there are no significant interaction effects, we also do not consider the covariate to have time-dependent effects. In such cases, we use only the main covariate in the model selection process (e.g., “packyears”). If both the main covariate and interaction terms are significant, we consider this covariate to have a time-dependent effect over landmark times and include both the main covariate and interaction in model selection (e.g., “radiation.ix” and “radiation.ix_1”).

For more thorough evaluation, readers might also consider how adding interaction change the model performance (i.e., calibration).

- formula <- "Hist(Time, event, LM) ~ packyears + packyears_1 + packyears_2 + cluster(ID)"
- check_td <- dynamic_lm(lmdata, as.formula(formula), "CSC")
- print(check_td, cause = 1)
- # packyears showed a significant effect, but packyears_1 and packyear_2 did not.
- formula <- "Hist(Time, event, LM) ~ packyears + packyears_1 + cluster(ID)"
- check_td <- dynamic_lm(lmdata, as.formula(formula), "CSC")
- print(check_td, cause = 1)
- # packyears showed a significant effect, but packyears_1 did not.
- # packyears does not have a time-dependent effect.
- formula <- "Hist(Time, event, LM) ~ radiation.ix + radiation.ix_1 + radiation.ix_2 + cluster(ID)"
- check_td <- dynamic_lm(lmdata, as.formula(formula), "CSC")
- print(check_td, cause = 1)
- # radiation.ix showed a significant effect, but radiation.ix_1 and radiation.ix_2 did not.
- formula <- "Hist(Time, event, LM) ~ radiation.ix + radiation.ix_1 + cluster(ID)"
- check_td <- dynamic_lm(lmdata, as.formula(formula), "CSC")
- print(check_td, cause = 1)
- # Both radiation.ix and radiation.ix_1 showed a significant effect.
- # radiation.ix has a time-dependent effect.
- # include both radiation.ix and radiation.ix_1 together in model development.
  - - 1. **Variable selection**

We fit univariate cause-specific Cox supermodels to identify covariates with <.1 liberal p-value to be considered as the potential predictors (features) of the model. A robust variance estimator was applied to account for dependency from multiple identical subjects in the stacked data.

- predictors <- c(fix_covs, vary_covs)
- for (i in predictors) {

formula <- paste("Hist(Time, event, LM) ~ ", i[[1]], "+ cluster(ID)")

supermodel_uni <- dynamic_lm(lmdata, as.formula(formula), "CSC")

print(supermodel_uni, cause = 1)

}

After examining the univariate models, we fit a full model that includes all features that showed p-value <.1 and additional features known to be associated with (or confounding factors for) SPLC risk in the literature. For the covariates with time-dependent effects identified in step (e), we added the interaction terms. Additionally, we included linear and quadratic landmark time (LM_1 and LM_2) variables to incorporate the smoothing effect over the stacked landmark datasets; this allows the fitted supermodel’s baseline hazard to depend on the landmark prediction time.

- formula <- "Hist(Time, event, LM) ~ age.ix + bmi + male + ph + ph_1 +

surgery.ix + cigday + packyears + quityears +

hist_AD + hist_LC + hist_NSCLC_NOS + hist_OTH + hist_SC +

hist_AD_1 + hist_LC_1 + hist_NSCLC_NOS_1 + hist_OTH_1 +

hist_SC_1 + radiation.ix + radiation.ix_1 + stage.ix +

stage.ix_1 + stage.ix_2 + LM_1 + LM_2 + cluster(ID)"

- supermodel_full <- dynamic_lm(lmdata, as.formula(formula), "CSC")
- print(supermodel_full)

For variable selection, we dropped one variable at each time, starting from the full model, by evaluating the interpretability of the estimated hazard ratios, the predictive performance metrics (discrimination, calibration, and brier score), and the statistical significance. Below is the final selected model selected. The dynamic cause-specific hazard ratios of the selected model are depicted in **Supplementary Table S2**. The covariates with time-dependent effects included IPLC radiotherapy, with its effect increasing over landmark times (**Supplementary Figure S2**).

- formula <- "Hist(Time, event, LM)~ male + ph + ph_1 +

packyears + quityears + hist_AD + hist_LC + hist_NSCLC_NOS +

hist_OTH + hist_SC + hist_AD_1 + hist_LC_1 + hist_NSCLC_NOS_1 +

hist_OTH_1 + hist_SC_1 + radiation.ix + radiation.ix_1 +

stage.ix + stage.ix_1 + stage.ix_2 + LM_1 + LM_2 + cluster(ID)"

- supermodel <- dynamic_lm(lmdata, as.formula(formula), "CSC", x = TRUE)
- print(supermodel)

Dynamic hazard ratios for the variables included in the selected model can be plotted.

- par(mfrow = c(2, 3))
- plot(supermodel,

logHR = FALSE,

covars = c("ph", "stage.ix", "hist_LC", "hist_SC", "radiation.ix"),

main = c("Prior history of cancer", "IPLC stage (Advanced)",

"IPLC histology (LC)", "IPLC histology (SC)",

"IPLC radiotherapy"),

xlab = "Risk Assessment Time \n (Landmark Prediction Time)",

ylab = "Adjusted csHR")

The 5-year predicted risk of developing SPLC among individual LC patients based on the selected model can be estimated using the following line.

- p <- predict(supermodel)
- predicted_risk <- p$preds
- summary(predicted_risk$risk)

An example of two patients’ dynamic 5-year SPLC risk is shown in **Figure 1B**.

- par(mfrow = c(1, 1))
- inds <- lmdata$data[lmdata$data$ID %in% c("ID2", "ID7"), ]
- plotrisk(supermodel, inds, format = "long", ylim = c(0, 0.2))

In **Figure 1B**, the dynamic trajectory of estimated 5-year SPLC risk among two example individuals are shown across different risk assessment times (Landmark times). With a 5-year predefined prediction window (w=5), the risk of developing SPLC in 5 years was estimated at each landmark time (i.e., risk assessment time or prediction time point across the horizontal coordinates. In this example, the 5-year risk of developing SPLC in patient P1 is 0.97% when predicted at IPLC diagnosis (landmark = 0), which is updated to 1.43% (when predicted at 1-year after IPLC diagnosis [landmark = 1]), 3.06% (when predicted at 2-year after IPLC diagnosis [landmark = 2]), and 4.38% (when predicted at 3-year after IPLC diagnosis [landmark =3]), at successive landmarks based on the most recent patient information seen till each landmark.

- - - 1. **Model evaluation**

To assess the predictive model performance, we create calibration plots and estimate the AUC and Brier score. Overall, the proposed model showed good performance (range of calibration slop [0.9-1.2]) across landmark times (**Figure 1A and Supplementary Figure S3**).

- par(mfrow = c(2, 2))
- cal <- calplot(list("CSC" = p), method = "quantile", q = 10,

ylim = c(0, 0.25), xlim = c(0, 0.25))

The AUC was >85% at baseline, which decreased over landmark times because most of the available data for model fitting came from the baseline in MEC. If patient features are updated and supplemented over time, the predictive accuracy could stay consistent.

- scores <- score(list("CSC" = p), cause = 1)
- print(scores)
- par(mfrow = c(1, 2))
- plot(scores)
  - - 1. **Model validation for generalizability**

An essential step in assessing the generalizability of prediction models is to validate the proposed models using internal validation in the bootstrapped resampled data or using external validation in the independent external data sources.

- ***Bootstrapped internal validation***

Bootstrapped internal validation prevents overfitting the training data and over-optimistic estimates. Bootstrap cross-validation can be performed where B models are trained on bootstrap samples either drawn with replacement of the same size as the original data or without replacement of size M. Models are then assessed in observations not in the bootstrap sample.

- par(mfrow = c(2, 2))
- cal <- calplot(list("CSC" = supermodel), method = "quantile", q = 10,

ylim = c(0, 0.25), xlim = c(0, 0.25),

split.method = "bootcv", B = 10)

- score(list("CSC" = supermodel), cause = 1, split.method = "bootcv", B = 10)
- ***External validation***
- # New stacked data with a LM column
- data(splc_test)
- table(splc_test$LM)
- # lms argument gives the landmark column
- par(mfrow = c(2,2))
- cal <- calplot(list("CSC" = supermodel), data = splc_test, lms = "LM",

method = "quantile", q = 10,

ylim = c(0, 0.25), xlim = c(0, 0.25))

- scores_external <- score(list("CSC" = supermodel), cause = 1,

data = splc_test, lms = "LM")

- print(scores_external)

## **[3] Simulation Methods**

In simulating a set of landmark datasets under competing risks, we extended the data-generating concept for a binary outcome in Austin et al., which utilizes an existing dataset for simulation that reflects the correlation among the variables observed in the dataset.^5^ In particular, we used the example dataset “*relapse*” (available and described in *dynamicLM*) to fit a landmark supermodel to obtain coefficients for each covariate $\beta\left( s \right)= \beta_{0}+ \beta_{1}s+ \beta_{2}s^{2}$ and for the baseline hazard $\theta\left( s \right)= \theta_{0}+\theta_{1}s$, and then generated data based on this model. The data-generating model includes the same covariates as in *relapse* and reflects the multivariate distribution observed. We simulated data as follows:

First, consider the following methodology (*Simulation A*) to simulate survival data without competing events:

1. Fit a Cox model to the given dataset (of covariates and outcome variable).
2. Calculate the linear combination of the parameters and covariate values for the event of interest using the model.
3. Using this linear combination vector as *a single covariate* and based on the hazard rates estimated from the Cox model, simulate an outcome time of the event for each person. We use the R package coxed, based on Harden and Kropko (2018)^6^ for this step.
4. Combine this new time-to-event outcome with the variables of the original dataset; this new data resembles the truth of the model while fixing the same correlation structure of the covariates.

Second, consider the following methodology (*Simulation B*) to simulate competing risks survival data:

1. Fit a cause-specific Cox model to the given dataset (of covariates and outcome variable).
2. For each Cox model that makes up the cause-specific Cox model, run steps A.2-A.3 to simulate an event time for each cause.
3. Set the outcome time as the minimum of the simulated times in B.2 and the event as the corresponding event.
4. Combine this new time and event outcome with the variables of the original dataset; this new data resembles the truth of the model while fixing the same correlation structure of the covariates.

Lastly, consider the following methodology to imitate the landmark model based on Simulation A and Simulation B:

1. Pick a set of landmark times, say $\left\{ s_{0},\ldots,s_{L} \right\}$
2. Initially, define $K$ as the set of all patients included in the original dataset to be used for simulation.
3. For the first landmark time, $s=s_{0}$,
   1. Simulate outcome times and events for each patient in $K$ as in *Simulation B* using steps B.2-B.4. To do so, fit the cause-specific Cox model defined by the landmark dependent hazard rate, $h_{j}\left( t | s \right)=h_{j0}\left( t \right)\exp\left( \theta_{j}\left( s \right) \right)$, for A.3 and the single linear combination vector created from the patient’s covariates and $\beta_{j}\left( s \right)$ for step A.2.
   2. Define $K_{s}$as the set of any patients with their simulated times greater than$s+w$ and mark them as censored at $s+w$. Only retain predictions for patients whose event time falls within the interval $[s,s+w)$.
4. For each remaining landmark times, $s\in\left\{ s_{1},\ldots,s_{L} \right\}:$
   1. Simulate outcome times and events for each patient in $K_{s-1}$ as in *Simulation C.3.a- C.3.b*. Any previously simulated times and events are to be ignored.

**[4] Landmark Model**

## **Overview**

The landmark model for survival data is built on the concept of risk assessment times (i.e., landmarks) that span risk prediction times of interest, using the information on the individuals who survived up to that given time point. In this approach, the dataset of the study cohort is transformed into multiple censored datasets based on a prediction window of interest and the predefined landmark times. A model is fit on the stacked super dataset (i.e., supermodel), and dynamic risk prediction is then performed by using the most up-to-date value of a patient's covariate values. The *dynamicLM* tool aims to provide a simple framework to perform dynamic $w$-year risk predictions (i.e., risk of developing the event of interest within $w$ years from the risk assessment) from landmark time points (*t*_LMs_), allowing for competing risks. Specifically, risk prediction for the next $w$ years is made at baseline (e.g., diagnosis) as well as at a later set of risk assessment times (“landmark prediction times”) after baseline (e.g., at 1, 2, and 3 years after diagnosis), where $w$is a fixed prediction window.

## **Landmarking**

Landmarking for risk prediction on survival data is a method for $w$-year dynamic risk prediction where an individual has a personalized risk prediction which is updated as new information is collected among those who survived at a given time for risk assessment. Traditionally, a separate Cox proportional hazards (PH) model is applied to each landmark dataset.^7^ Predictions can then be made at each landmark time point. The landmark supermodel combines these models by introducing smoothing to permit risk prediction at any landmark.^8^


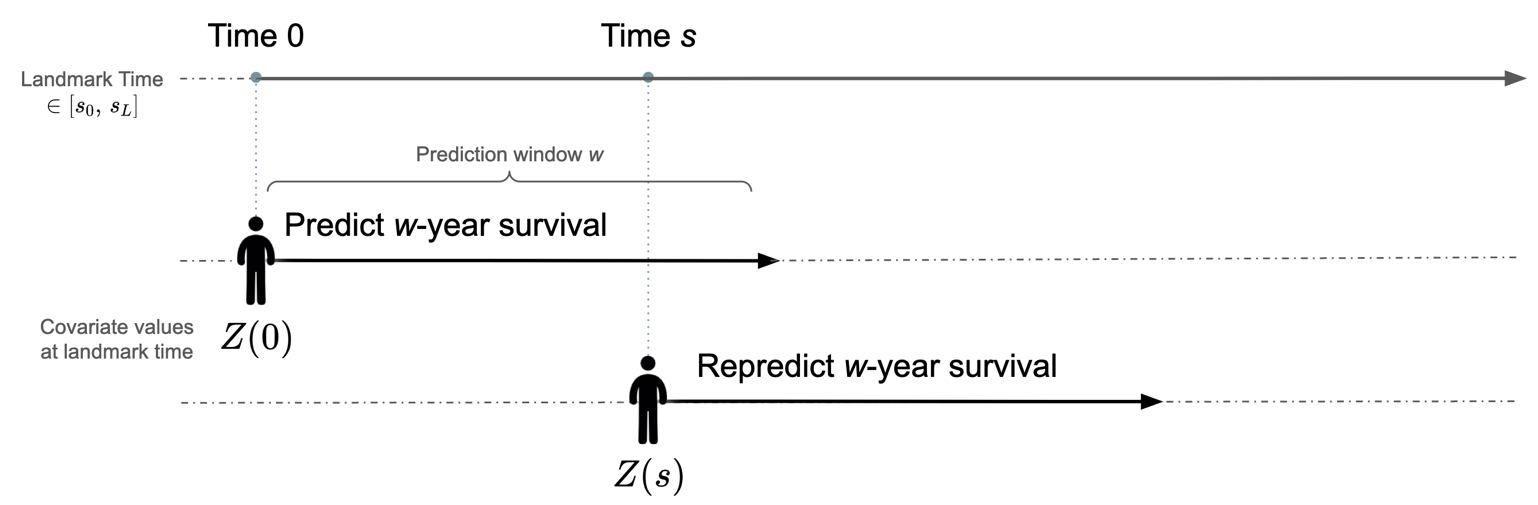
Landmarking was first introduced as a concept by Anderson et al. (1983)^9^, was adapted to dynamic prediction for survival by van Houwelingen (2007)^10^, and then used in the context of time-dependent covariates by van Houwelingen and Putter (2008)^11^. It was then conceptualized for competing risks by Nicolaie and van Houwelingen (2012)^8^. The following framework is explained in further detail in van Houwelingen (2007)^10^ and Nicolaie and van Houwelingen (2012)^8^.

## **Sliding landmark model and extension to competing risks**

Let $w$ be the prediction window of interest. We aim to create a model to estimate $w$-year risk at a landmark time $s$, knowing an individual’s covariates at $s$, $Z(s)$, and conditional on being alive at $s$. To create the landmark model, risk prediction times of interest are first partitioned into different landmarks $\{s_{0},\ldots,s_{L}\}$. The *sliding landmark dataset* is created for each landmark $s$, using only the data of individuals at risk (i.e., not censored or having experienced an event) with administrative censoring applied after $s+w$. For a time-dependent covariate, the dataset contains the most recent covariate value of the patient $Z(s)$, which is considered as a fixed variable.

Suppose that individuals can experience one of $C$ types of failure ('causes'). Competing risk analysis accounts for the probability of other causes of failure, by estimating the probability of a specific event while considering the presence of competing events. Each event (‘cause’) is modelled through a cause-specific hazard.

To create the sliding landmark model, a separate cox model is fit to each dataset by maximizing the Cox partial likelihood to find the parameters $\beta\left( s \right)$: leading to a conditional hazard of the following form:

- Standard survival analysis:

$h\left( t | Z\left( s \right),s \right)=h_{0}\left( t | s \right)\exp\left( Z\left( s \right)\beta\left( s \right) \right)$ $s\leq t\leq s+w$

- Under competing-risk, for cause $j$

$h_{j}\left( t | Z\left( s \right),s \right)=h_{j0}\left( t | s \right)\exp\left( Z\left( s \right)\beta_{j}\left( s \right) \right)$ $s\leq t\leq s+w, j\in C$

In summary, the sliding landmark model consists of different models for each landmark, and predictions can only be made at these predefined landmarks.

## **The landmark supermodel**

At a high-level, the landmark supermodel introduces smoothing between the separate sliding landmark models, to create one model that provides $w$-year prediction at any $s\in[s_{0},s_{L}]$.

A super dataset is built to create the landmark supermodel:

1. Fix $w$, a prediction horizon/window, and partition risk prediction times of interest into different landmarks $\{s_{0},\ldots,s_{L}\}$.
2. Build a landmark data set for each $s_{i}$ as before (i.e., with left truncation at $s_{i}$, right administrative censoring after $s_{i}+w$, and using the covariate values $Z(s_{i})$)
3. Stack these data sets to create a “super prediction data set.”

A Cox (or cause-specific Cox) model is trained on the super dataset. To account for covariate landmark-varying effects, the regression coefficients depend smoothly on $t_{LM}=s$ (modelled linearly), i.e., $\beta\left( s \right)= \sum_{k} \beta_{k}f_{k}(s)$ for some functions $f_{k}(s)$. The default in our implementation is

$$\beta\left( s \right)= \beta_{0}+ \beta_{1}s+ \beta_{2}s^{2}$$

Note that the regression parameters depend on prediction time, not event time! The baseline hazard also depends on $s$, and this can be modelled by: $h_{0}\left( t | s \right)=h_{0}\left( t \right)\exp(\theta\left( s \right))$. The default in our implementation is

$$\theta\left( s \right)= \theta_{0}+\theta_{1}s$$

Such a model is then fit on the super dataset, which leads to the hazard:

$$h_{j}\left( t | Z\left( s \right),s \right)=h_{j0}\left( t \right)\exp\left( \theta_{j}\left( s \right)+ \beta_{j}\left( s \right)Z(s) \right)$$

In summary, the main effects for the landmark time $s$ is modeled by $\theta(s)$ and the interaction of $s$ with the covariates is modeled by $\beta(s)$. Dependence between entries needs to be accounted for, for example, by using a robust sandwich estimator, as the same patient appears multiple times in the super dataset. In practice in R, a cluster argument is used when specifying the model formula: “Surv(time, event) ~ variable + cluster(ID)”, where “ID” denotes the subject identifier.

## **Prediction**

The cumulative hazard is defined as $H\left( s+w | Z\left( s \right),s \right)=\int_{s}^{s+w} h\left( t | Z\left( s \right),s \right)dt$, and is used for prediction. Under the Cox model (time-to-event data), predictions are made as follows, where $S$ is survival and $F$ is cumulative incidence:

$$S\left( s+w | Z\left( s \right),s \right)=\exp\left( -\int_{s}^{s+w} h\left( t | Z\left( s \right),s \right)dt \right)$$

$$F\left( s+w | Z\left( s \right),s \right)=1- S\left( s+w | Z\left( s \right),s \right)$$

Under competing risks, all cause-specific hazards are considered, with coefficients from each of the cause-specific Cox models, $\beta_{j}\left( s \right)= \beta_{j0}+ \beta_{j1}s+ \beta_{j2}s^{2}$ and $\theta_{j}\left( s \right)= \theta_{j0}+\theta_{j1}s$. To compute survival, sum over the cumulative cause-specific hazards:

$$S\left( s+w | Z\left( s \right),s \right)=\exp\left( -\int_{s}^{s+w} \sum_{j=1}^{C} h_{j}\left( t | Z\left( s \right),s \right)dt \right)$$

$$=\exp\left( -\sum_{s<t_{i}\leq s+w} \sum_{j=1}^{C} h_{j}\left( t_{i} | Z\left( s \right),s \right) \right)$$

(where $t_{i}$ are event times in the original dataset)

Cause-specific cumulative incidence considers chance of failure from cause $j$ in each interval conditional on surviving to that point

$$F_{j}\left( s+w | Z\left( s \right),s \right)=\int_{s}^{s+w} h_{j}\left( t | Z\left( s \right),s \right)S\left( t | Z\left( s \right),s \right)dt$$

$$=\sum_{s<t_{i}\leq s+w} h_{j}\left( t_{i} | Z\left( s \right),s \right)S\left( t_{i} | Z\left( s \right),s \right)$$

Note that for the sliding landmark model, $h_{j}\left( t | Z\left( s \right),s \right)=h_{j0}\left( t | s \right)\exp\left( Z\left( s \right)\beta_{j}\left( s \right) \right)$and for the supermodel, $h_{j}\left( t | Z\left( s \right),s \right)=h_{j0}\left( t \right)\exp\left( \theta_{j}\left( s \right)+ \beta_{j}\left( s \right)Z(s) \right)$.

## **Model performance**

Current methods to evaluate dynamic risk prediction models involve evaluating calibration and discrimination at each landmark point individually. *Dynamic time-dependent AUC* (AUCt)^12^ measures discrimination. As the landmark model performs $w$-year risk prediction, discrimination between those who incur events before and after $w$-year prediction is of interest. Simply put, AUCt is the percentage of correctly ordered markers when comparing a case (those who incur the event of interest before the window) and a control (those who do not)- – i.e., those who incur the primary event within the window $w$ after prediction and those who do not. More formally, AUCt is defined as the ratio of the estimated probability of observing a pair of a case and a control with ordered markers over the estimated probability of observing a pair with a case and a control. Let the $t$-year risk prediction (marker) of an individual $i$ with covariate information $Z_{i}(s)$ at time $s$ be labeled $M_{i}(s,t)$ and let $M_{i}\left( 0,t \right):=M_{i}$

Under time-to-event data, time-dependent AUC is defined as:

$$AUC\left( t \right)=P\left( M_{i}\left( 0,t \right)>M_{j}(0,t) \right| T_{i}\leq t,T_{j}>t)$$

The cases are the individuals $i$ who incur an event before $t$, and controls are those that survive past $t.$ Under competing-risk

$$AUC\left( t \right)=P\left( M_{i}\left( 0,t \right)>M_{j}\left( 0,t \right) \right| T_{i}\leq t,\eta_{i}=1,\left\{ T_{j}>t \right\}\mathrm{or}\{T_{j}\leq t, \eta_{j}\neq1\})$$

The cases are the individuals $i$ with event before $t$, and controls are those that survive past $t$ or incur a competing risk before $t$. Note that $\eta_{i}$ is the event that $i$ undergoes.

When extending to landmark times, the cases are those that experience the event in $\left[ s,s+w \right]$ and controls survive past them. The time-to-event and competing-risk definitions are:

$$AUC\left( s,t \right)=P\left( M_{i}(s,t)>M_{j}(s,t) \right| {s\leq T}_{i}\leq t,T_{j}>t)$$

$$AUC\left( s,t \right)=P\left( M_{i}\left( s,t \right)>M_{j}\left( s,t \right) \right| {s\leq T}_{i}\leq t,\eta_{i}=1,\left\{ T_{j}>t \right\}\mathrm{or}\{{s\leq T}_{j}\leq t, \eta_{j}\neq1\}) )$$

When performing dynamic risk prediction using landmarking, $AUC\left( s,s+w \right)$ is calculated for each landmark $s$ used in prediction.

It should be noted that the C-index is not appropriate as it assesses the order of the event times and not the order of the event status at the prediction horizon.

$$\boldsymbol{C} = P\left( M_{i}>M_{j} \right|T_{i}<T_{j})$$

*Dynamic time-dependent Brier^12^* (BSt) is also provided. The Brier Score summarizes the squared difference between event indicators $\eta_{i}$and risk estimates $M_{i}.$Its formulation combines definitions of the expected Brier score for competing risks^13^, which is the average squared difference between the primary event markers at the end of the prediction horizon and the absolute risk estimates by that time point, and for dynamic prediction^14^ leading to

$$BS\left( s,t \right)\mathbb{= E}\left[ \left( 1\left\{ s\leq T<s+w, \eta_{i}=1 \right\}-M\left( s,t \right) \right)^{2} \right|T>s]$$

When performing dynamic risk prediction using landmarking, $BS(s,s+w)$ is calculated for each landmark $s$ used in prediction.

*Calibration plots* are important to assess the agreement between predictions and observations in different percentiles of the predicted values. These can be plotted for each of the landmarks used for prediction.

# **4. References**

1. Aredo JV, Luo SJ, Gardner RM, et al. Tobacco Smoking and Risk of Second Primary Lung Cancer. *J Thorac Oncol.* 2021;**16**:968-979.

2. Choi E, Sanyal N, Ding VY, et al. Development and Validation of a Risk Prediction Model for Second Primary Lung Cancer. *J Natl Cancer Inst.* 2022;**114**:87-96.

3. Martini N, Melamed MR. Multiple primary lung cancers. *J Thorac Cardiovasc Surg.* 1975;**70**:606-612.

4. Choi E, Lam VT, Aredo JA, et al. Abstract 3445: Long term effect of radiotherapy on risk of second primary lung cancer and overall mortality among lung cancer patients. *Cancer Research.* 2022;**82**:3445-3445.

5. Austin PC, Harrell FE, Jr., Steyerberg EW. Predictive performance of machine and statistical learning methods: Impact of data-generating processes on external validity in the "large N, small p" setting. *Stat Methods Med Res.* 2021;**30**:1465-1483.

6. Harden JJ, Kropko J. Simulating Duration Data for the Cox Model. *Political Science Research and Methods.* 2019;**7**:921-928.

7. van Houwelingen H, Putter H. Dynamic prediction in clinical survival analysis: CRC Press 2011.

8. Nicolaie MA, van Houwelingen JC, de Witte TM, Putter H. Dynamic prediction by landmarking in competing risks. *Stat Med.* 2013;**32**:2031-2047.

9. Anderson JR, Cain KC, Gelber RD. Analysis of survival by tumor response. *J Clin Oncol.* 1983;**1**:710-719.

10. Van Houwelingen HC. Dynamic prediction by landmarking in event history analysis. *Scandinavian Journal of Statistics.* 2007;**34**:70-85.

11. van Houwelingen HC, Putter H. Dynamic predicting by landmarking as an alternative for multi-state modeling: an application to acute lymphoid leukemia data. *Lifetime Data Anal.* 2008;**14**:447-463.

12. Blanche P, Proust-Lima C, Loubère L, et al. Quantifying and comparing dynamic predictive accuracy of joint models for longitudinal marker and time-to-event in presence of censoring and competing risks. *Biometrics.* 2015;**71**:102-113.

13. Schoop R, Beyersmann J, Schumacher M, Binder H. Quantifying the predictive accuracy of time-to-event models in the presence of competing risks. *Biom J.* 2011;**53**:88-112.

14. Schoop R, Graf E, Schumacher M. Quantifying the predictive performance of prognostic models for censored survival data with time-dependent covariates. *Biometrics.* 2008;**64**:603-610.
